# Supplementary material for: Phase I/II Trial of Urokinase Plasminogen Activator-Targeted Oncolytic Newcastle Disease Virus for Canine Intracranial Tumors
Source: Cancers (Basel). 2024 Jan 29;16(3):564. doi: 10.3390/cancers16030564 (PMC10854777; doi:10.3390/cancers16030564)
Supplement: Supplementary file 1 [file cancers-16-00564-s001.zip › cancers-2819467-supplementary.pdf]

Supplemental Material

Phase I/II Trial of Urokinase Plasminogen Activator-Targeted Oncolytic Newcastle Disease Virus for Canine Intracranial Tumors

John H. Rossmeisl, Jamie N. King, John L. Robertson, James Weger-Lucarelli and Subbiah Elankumaran

Supplemental Table S1. Canine Subject and Tumor Characteristics.

Dog	rLAS-uPA Dose Cohort	Breed	Age (years)	Sex	BW (kg)	Clinical Signs	Baseline KPS	Tumor Location	Tumor Histology	Baseline TTV (cm^3)	uPAR IHC Score
1	2 x 10^4 pfu/ml	Mixed breed	11	FS	39	Seizures, Circling	75	Parasagittal	NA- Presumptive Meningioma	4.23	NA
2	2 x 10^4 pfu/ml	Spitz	12	FS	9	Seizures	90	Cystic olfactory	NA-Presumptive Meningioma	1.09	NA
3	2 x 10^4 pfu/ml	Border Terrier	5	MN	7	Seizures	90	Cerebral convexity	Grade II Meningioma	2.78	3
4	2 x 10^5 pfu/ml	Jack Russell Terrier	8	MN	8	Chiasmal blindness	90	Parasellar	NA- Presumptive Meningioma	1.13	NA
5	2 x 10^5 pfu/ml	Boxer	12	MN	31	Seizures, thalamocortical visual deficit	80	Parasagittal	Grade I Meningioma	6.71	2
6	2 x 10^5 pfu/ml	Irish Setter	11	MN	23	Seizures	80	Falcine	Grade I Meningioma	5.69	2
7	2 x 10^7 pfu/ml	German Shepherd	10	FS	34	Seizures	70	Cerebral convexity	NA- Presumptive Meningioma	8.14	NA
8	2 x 10^7 pfu/ml	Boston Terrier	8	FS	11	Seizures, behavior change	70	Thalamic	High-grade astrocytoma	2.15	6
9	2 x 10^7 pfu/ml	Mixed breed	9	FS	13	Unilateral masticatory muscle atrophy	90	Extra-axial mass at level of pons	Schwannoma	1.62	3
10	2 x 10^9 pfu/ml	Chow	7	MN	22	Central vestibular dysfunction	70	Tentorial	Grade II Meningioma	1.96	3
11	2 x 10^9 pfu/ml	Mixed breed	7	MN	27	Seizures, Hemi-inattention	80	Cerebral convexity/ En-plaque	Grade I Meningioma	1.85	3
12	2 x 10^7 pfu/ml	Fox Terrier	9	FS	9	Unilateral cavernous sinus syndrome and masticatory muscle atrophy	70	Extra-axial mass at level mesencephalon/pons	Malignant Peripheral Nerve Sheath Tumor	1.29	5
13	2 x 10^7 pfu/ml	Rottweiler	10	MN	42	Seizures	80	Parasagittal	Grade I Meningioma	8.23	2
14	2 x 10^7 pfu/ml	Golden Retriever	11	FS	26	Seizures	90	Olfactory	Grade II Meningioma	5.12	4
15	2 x 10^7 pfu/ml	French Bulldog	6	FS	13	Seizures, circling	70	Hemispheric	High-grade oligodendroglioma	3.29	5
16	2 x 10^7 pfu/ml	Golden Retriever	5	MN	26	Paradoxical central vestibular syndrome	70	Cerebelloponto-medullary angle	Choroid plexus papilloma	2.24	6
17	2 x 10^7 pfu/ml	Boxer-X	7	MN	2146	Seizures	90	Hemispheric	Low-grade oligodendroglioma	3.78	3
18	2 x 10^7 pfu/ml	Am Staff Terrier	5	FS	38	Circling, head pressing	70	Lateral ventricle	Choroid plexus carcinoma	5.54	5
19	2 x 10^7 pfu/ml	Boston Terrier	9	MN	10	Seizures	90	Hemispheric	High-grade oligodendroglioma	2.67	4
20	2 x 10^7 pfu/ml	Beagle	10	MN	11	Seizures	90	Parasellar	Grade I Meningioma	1.96	2

Table S1 Key

IHC= Immunohistochemistry; NA= not applicable

KPS= Karnofsky Performance Score
NA= Data not available
TTV= T2 Total Tumor Volume

Supplemental Table S2. Canine Subject Off-protocol Clinical and Follow-up Data.

Dog	rLAS-uPA Dose Cohort	Tumor Histology	Pre-Enrollment Treatment	Post-trial Treatment	Follow-up Duration (Days from Trial Enrollment)	Outcome
**1**	2 x 10^4 pfu/ml	NA- Presumptive Meningioma	None	None	46	Death SD- Status Epilepticus
**2**	2 x 10^4 pfu/ml	NA-Presumptive Meningioma	None	None	242	Death PD
**3**	2 x 10^4 pfu/ml	Grade II Meningioma	Surgery	Investigational, Surgery, Radiotherapy	789	Death PD
**4**	2 x 10^5 pfu/ml	NA- Presumptive Meningioma	None	None	623	Death Presumed PD
**5**	2 x 10^5 pfu/ml	Grade I Meningioma	Surgery	None	265	LTF- Unknown
**6**	2 x 10^5 pfu/ml	Grade I Meningioma	Surgery	None	419	Death PD
**7**	2 x 10^7 pfu/ml	NA- Presumptive Meningioma	None	None	164	Death Presumed PD
**8**	2 x 10^7 pfu/ml	High-grade astrocytoma	Temozolomide	Radiotherapy	316	Death Presumed PD
**9**	2 x 10^7 pfu/ml	Schwannoma	Surgery	None	224	LTF- Unknown
**10**	2 x 10^9 pfu/ml	Grade II Meningioma	Investigational	None	275	Death UD
**11**	2 x 10^9 pfu/ml	Grade I Meningioma	Investigational	Surgery	195	LTF- Unknown
**12**	2 x 10^7 pfu/ml	Malignant Peripheral Nerve Sheath Tumor	Surgery	None	221	Death Presumed PD
**13**	2 x 10^7 pfu/ml	Grade I Meningioma	Hydroxyurea	Surgery, Investigational	562	Death PD
**14**	2 x 10^7 pfu/ml	Grade II Meningioma	Surgery	None	271	Death PD
**15**	2 x 10^7 pfu/ml	High-grade oligodendroglioma	Investigational	Investigational	340	Death PD
**16**	2 x 10^7 pfu/ml	Choroid plexus papilloma	Radiotherapy	None	303	LTF- Unknown
**17**	2 x 10^7 pfu/ml	Low-grade oligodendroglioma	Surgery	Investigational	635	Death SD- Seizures
**18**	2 x 10^7 pfu/ml	Choroid plexus carcinoma	Radiotherapy	None	107	Death PD
**19**	2 x 10^7 pfu/ml	High-grade oligodendroglioma	Lomustine	None	298	Death PD
**20**	2 x 10^7 pfu/ml	Grade I Meningioma	Hydroxyurea	Surgery	528	Death PD

Table S2 Key
LTF= Lost to follow-up
PD= Progressive disease (confirmed with MRI and/or necropsy examination)
Presumed PD= Presumed progressive disease with intensification of pre-existing tumor-related neurologic signs or development of new neurologic signs
SD= Stable disease
UD= Disease unrelated to tumor

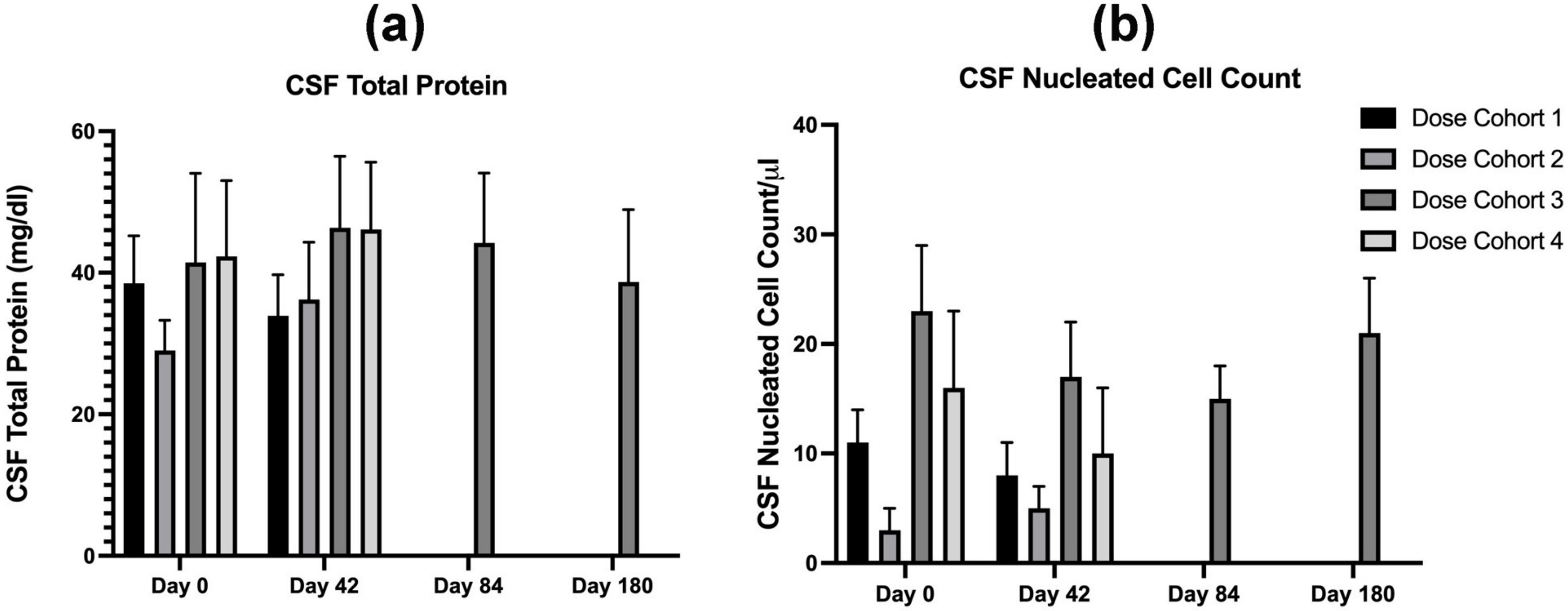

Figure S1. Cerebrospinal Fluid (CSF) Total Protein and Nucleated Cell Counts in Canine Subjects. Legend- rLAS-UPA treatment was not associated with statistically significant changes in CSF total protein (**a**) or nucleated cell counts (**b**) in canine subjects.
